# Effects of a school-based intervention on levels of anxiety and depression: a cluster-randomized controlled trial of the MindPower program in ten high schools in Norway

**DOI:** 10.1186/s40359-022-00721-y

**Published:** 2022-01-24

**Authors:** Gry Anette Sælid, Nikolai Olavi Czajkowski, Leif Edvard Aarø, John Roger Andersen, Thormod Idsøe, Miguel Delgado Helleseter, Arne Holte

**Affiliations:** 1 Division of Mental and Physical Health, Norwegian Institute of Public Health, Marcus Thranes Gate 6, 0473 Oslo, Norway; 2 Department of Psychology, University of Oslo, Forskningsveien 3A Harald Schjelderups hus, 0373 Oslo, Norway; 3 Department of Health and Caring Sciences, Western Norway University of Applied Sciences, Svanehaugvegen 1, 6812 Førde, Norway; 4 Norwegian Center for Child Behavioral Development, Essendrops Gate 3, Postboks 7053 Majorstuen, 0306 Oslo, Norway; 5 Institute for Global Research, California State University Channel Islands, One University, Drive Camarillo, CA 93012 USA

**Keywords:** Mental health promotion, Prevention, Anxiety, Depression, Universal, School, Adolescent, CWD

## Abstract

**Background:**

The previous decades have shown increased symptoms of depression and anxiety among adolescents. To promote mental health and reduce mental illness, the government of Norway has, as in other countries, pledged that all schools must incorporate life-skills education. We report results from an evaluation of MindPower, a modification of the Coping With Depression (CWD) course, delivered universally in the classroom to secondary high school students, aged 15–16 years, in one county in Norway. The aim of the study was to evaluate the effect of MindPower on symptoms of depression and anxiety.

**Methods:**

We utilized a two-groups` delayed intervention design where 110 first year high school classes were randomized into one of two intervention groups (IG1 and IG2). IG1 participated in MindPower while IG2 served as a control group for four months until the intervention started also in this group. IG1 and IG2 responded to questionnaires before and after the eight weeks course, at the start of the first and the second booster session, and at the five months follow up. Questionnaires, including online versions of the Hopkins Symptom Checklist (SCL-8) and the Reynolds Adolescent Depression Scale (RADS-2:SF), were administered to 1673 out of a total of 2384 students. SCL-levels were also compared with those from a large population study (UngData).

**Results:**

According to mixed model analyses, SCL-8 and RADS-2:SF showed significant baseline differences between IG1 and IG2. In IG1 and IG2, both SCL-8 and RADS-2:SF showed a small but significant increase in mean scores throughout the study period, with markedly lower mean scores among boys. The SCL-levels were first lower for both girls and boys and then after the completion of MindPower the SCL-levels, equal to the SCL-levels in UngData.

**Conclusions:**

No effects of the intervention were found. This large universal school-based trial suffered from considerable drop-out of participants. Experiences from implementation and evaluation of universal mental health promotion and preventive school interventions are thoroughly discussed, including, preparation, resources, support, time, realistic expectations, teacher selection and training, implementation, research designs and more. Several empirically based, practical advices are presented.

*Clinical Trial registration*

27/08/2018. Registration number NCT03647826.

## Background

Between 10—20 percent of children and adolescents in the western world report mental health problems [1]. During the past decade, increasing levels of mental distress and common mental disorder have been reported across countries, especially among young girls [2].

To meet these challenges, initiatives to promote mental health and reduce mental illness in educational settings are at the forefront in many countries [3]. Schools are well positioned to promote mental health and prevent mental illness due to the amount of time all children and adolescents spend in this environment [4].

A number of studies show that effects sizes in evaluations of universal interventions tend to be rather small [5]. Expecting program effects to reach 0.50 or 0.80 in the field of universal prevention programs might be unrealistic. Since universal programmes reach large population segments, these small effects may still be important from a public health perspective [6]. This is an important premise for realistic evaluation of such programmes [7]. Systematic reviews and meta-analyses show positive short-term effects of school-based initiatives aimed at enhancing across a wide range of outcomes [8], including children’s life-skills [9], resilience [10], mindfulness (11, 12, social and emotional competence [13], and to reduce mental distress and prevent common mental disorder [14–18]. In addition, numerous guidelines and policies on how to integrate health and education in schools have been published [19–21].

In this study, we examine effects of the MindPower program. Our study is the first evaluation of MindPower. MindPower is a universal adaption of the Coping With Strain (CWS) course, which is a modification of the Coping With Depression (CWD) course. CWD/CWS interventions have been tested for 30 years in several settings [22, 25], ranging from treatment facilities for depression [26], programmes targeting adolescents [27], to workplaces [28, 29]. However, most of these initiatives have targeted groups with an elevated risk. These studies generally report high effect sizes, and some suffer from high drop-out, e.g. CWS in workplaces. Most studies evaluate programmes which take place in clinical settings. This stands in some contrast to the established recommendation that when a health condition is widespread in a population, universal interventions should be preferred [30–33]. Nowadays, most interventions are very similar to the CWD in that they use the same modules, but without referring to the CWD. To our knowledge, however, the only universal version of the CWD-family of courses to date is the 30 years old version of Clark et al. [22].

The aim of this cluster randomized controlled trial is to test whether MindPower has positive effects on aspects of students’ mental health. Our hypothesis is that MindPower will prevent and reduce symptoms of depression and anxiety. We utilize a two-group design with an early intervention in one group and a delayed intervention in the second one. The delayed intervention group will serve as control until the intervention starts also in this group. Relative decreases in symptom levels during interventions when compared across groups as well as with trends before and after interventions are indicative of a programme effect. MindPower sample estimates are compared with statistics based on a large, representative population study (UngData).

## Methods

### Study design

**Fig. 1:**
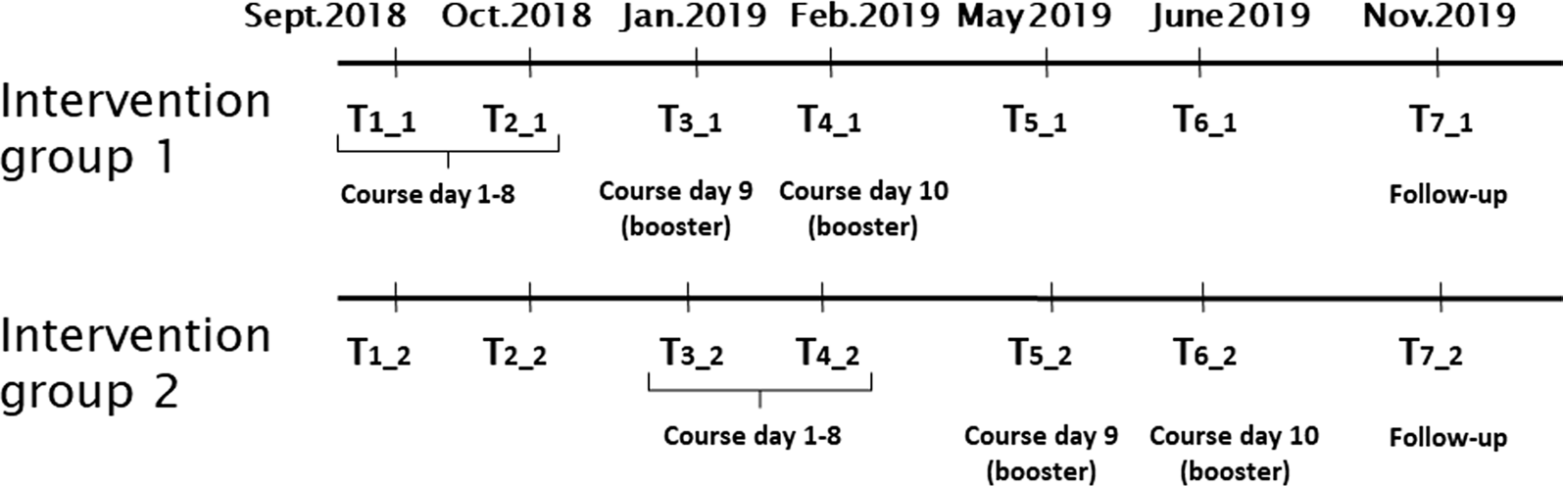
Study design

We used a parallel design, of a two-group delayed intervention design, in which data were collected at the same seven timepoints in both groups. Allocation ratio was 1:1. The study design is illustrated in Fig. 1. Each class at the participating schools were randomly assigned to one of the two intervention groups. The students in both groups completed questionnaires six times at school (T1-T6), and the seventh and final time at follow up at home (T7).

Intervention group 1 (IG1) was assessed when they started MindPower at time point 1 (T1) in September 2018, and eight weeks later at the last day of the program, at T2. They were then assessed at two booster sessions at T3 and T4, two and four months after the end of the course at T2.

Intervention group 2 (IG2) was assessed at T1 and T2, and when they started with MindPower at T3, in January 2019, five months after the start of IG1. Eight weeks later, at the last day of their program, they were assessed again at T4. They were then assessed at two booster sessions at T5 and T6, two and four months after the end of their course at T4.

The final data collection in both groups was T7, which took place in November 2019.

### Sample size and power

Our power calculations were based on the following assumptions: The difference between the groups would correspond to an effect size of 0.30, statistical significance would be obtained with p < 0.05, the power was set to 0.80, the size of the clusters 20, and the intraclass correlation (ICC) was assumed to be 0.05. The number of students needed was estimated to be 680. The power calculation was carried out with the University of Aberdeen Sample Size Calculator [28]. The assumed ICC is based on analyses of a distress scale used in the Norwegian part of the Health Behaviour in School Children study (HBSC) [34, 35].

### Samples

The MindPower sample consisted of 110 first year high school classes at ten high schools in Østfold county in South Eastern Norway. All the 15–16 years old students were invited to complete an online questionnaire which contained measures of depression and anxiety, and several other measures. The questionnaires were completed in the classroom setting during ordinary school hours.

The study only comprised schools that not already had started to use the MindPower program. No participants were excluded from the study at any time point, as this study evaluates a real-life scenario (tuition of MindPower) in classrooms. Our aim was to send invitations by e-mail to all 2384 students in the 110 clusters. However, a large number of e-mail accounts turned out to be invalid. As a consequence, the questionnaire was administered to 1673 students. Among these, 933 (55.8%) students completed the questionnaire at least once: 298 girls in IG1; 242 boys in IG1; 210 girls in IG2; 183 boys in IG2. Over time, fewer and fewer students responded. At the last measurement in total 81 participants responded (Table 1). Students who were unable to respond because they had not received the questionnaires or did not want to participate in the study, read a book or did homework while the rest of the class completed the questionnaire.

**Table 1:** UngData and MindPower sample. SCL-8 and RADS-2:SF in IG1 and IG2

		T1	T2	T3	T4	T5	T6	T7
UngData schools that participated in MindPower. SCL-8	Mean = 1.99							
	N = 2391							
	SD = 0.783							
UngData schools that did not participate in MindPower. SCL-8	Mean = 1.98							
	N = 18992							
	SD = 0.776							
*SCL-8*								
MindPower: IG1	Mean	1.76	1.88	1.88	1.88	1.97	2	2.01
	N	545	557	450	357	170	183	50
	SD	0.73	0.8	0.83	0.86	0.92	0.93	0.84
MindPower: IG2	Mean	1.89	1.86	1.9	2	1.95	1.86	2.09
	N	396	380	536	376	317	234	31
	SD	0.79	0.8	0.8	0.83	0.86	0.82	0.74
*RADS:SF-2*								
MindPower:IG1	Mean	18.83	19.96	20.09	20.31	21.14	21.15	20.88
	N	548	567	458	381	179	187	52
	SD	6.2	6.56	6.67	7.51	7.64	7.63	6.77
MindPower: IG2	Mean	19.7	19.48	20.1	20.28	20.19	19.92	21.63
	N	401	389	543	395	326	243	32
	SD	6.28	6.74	7.02	6.87	7.17	7.16	6.55

A comparison sample, UngData, was utilized in this study to compare the anxiety and depression levels from the MindPower sample. UngData is conducted every year in most of the municipalities in Norway. Children and adolescents between 10 and 18 of age complete a questionnaire about friends, parents, school, community, leisure activities, and physical and mental health. Additionally, for high school students, the questionnaire has questions about sexuality, substance abuse, violence and other risk behavior. Like MindPower, students complete the UngData questionnaire in the classroom setting during ordinary school hours. In the current study, only data from first year high school students were included. Norwegian Social Research (NOVA) at the Oslo Metropolitan University is responsible for the national coordination while the regional Drug and Alcohol Competence Centers are responsible for collecting the data at the municipal level.

The data from UngData used in this study, were collected mainly in June 2019. However, not all the high schools in the UngData-sample responded in June 2019. In the total UngData sample, respondents between 2017 and 2019 have been included. The total UngData sample consisted of 18992 students; 9423 boys and 9414 girls. Response rate was 75%. Of these 2391 students, 1227 boys and 1160 girls, came from the same schools as those included in the MindPower study.

Both MindPower groups in the study eventually receive the MindPower course (weeks 1–8 in IG1, and 16–24 in IG2). However, if we compare MindPower vs the UngData study, we are not able to get the longitudinal information about the comparison between IG1 and IG2. The delayed intervention design in the study is registered in the protocol and made specifically to compare IG1 and IG2. IG2 serves as control group or comparison group during the first 8 weeks of the study period. Furthermore, none of the groups can be regarded as control group only, and therefore we prefer to use the terms IG1 and IG2.

### Randomization

The random sequence specifying allocation into the two intervention groups was generated using the statistical computing platform R. An initial random seed was chosen based on the system clock at the time of allocation (1, 525, 868, 496), and a sequence of random numbers was generated using the runif function. Each school participating in the study offered a number of different fields of studies (FOS), and the number of classes in each field ranged from 1 to 7. Randomization was stratified by school ID and FOS, such that half of the classes within each FOS at a given school were randomly assigned to each of the two intervention groups. Allocation of classes into the intervention groups was known by the school in advance.

### Measures

Symptoms of depression and anxiety were measured in both MindPower and UngData by Symptom Check List—8 (SCL-8). SCL-8 is a short form of The Hopkins Symptom Checklist 90. It contains five items assessing anxiety and three assessing depression [36]. SCL is scored on a 4-point scale with response options 1 – not at all, 2 – a little bit, 3 – quite a bit and 4 extremely [37]. SCL-8 has previously demonstrated high validity, as well as a reliability of 0.91 [36]. In MindPower, SCL-8 had a Cronbach’s α across measurement waves ranging between 0.92—0.95. In UngData, SCL-8 had an α of 0.91.

The threshold values on the SCL-instrument indicate a mental health problem when the score is above 1.75, 1.85, and 2.0, respectively for SCL-25, SCL-10 and SCL-5 [32]. For adolescents, a cut-off point of 3.00 has been proposed to indicate severe symptoms of common mental health problems [37–40]. This cut-off point has been used by UngData in their reports [41]. We have chosen to follow this convention, applying a cut of point of 3.00 to indicate severe mental health problems, while a score of less than 1.95 (1.85/2.00) is regarded as having no or minimal levels of depression or anxiety for SCL-8.

In MindPower, symptoms of depression were also measured with Reynolds Adolescent Depression Scale (RADS-2:SF). RADS-2:SF is a short form of the 30-item RADS-2, containing 10 items with response categories ranging from 1–4. Total scores range from 10 to 40. From 10 to 25 represents a normal range of symptom endorsement, and higher scores indicate clinical levels [42]. Studies have found Cronbach’s alpha to range from 0.91 to 0.94 [43]. In MindPower, RADS had a Cronbach’s α across measurement waves ranging from 0.88 to 0.93. RADS-2:SF is included in the study because of the assumption of increased validity, as RADS-2:SF is adapted particularly to this population and is assumed to be sensitive to change [42].

The questionnaire used in MindPower also included measures on positive mental health, such as self-efficacy, self-esteem, quality of life, self-control and wellbeing at school. Some of these measures, along with the two questions that the students reported on fidelity will be reported on elsewhere [44].

Background variables were gender, age and the ten high schools.

In MindPower, data collections among participants were carried out at all seven time points. From UngData only one measurement time point was included.

### Intervention and procedure

MindPower is a group-based cognitive behavioral intervention. The aim is to give adolescents an understanding of what mental health is, and to train their abilities to cope with the strains of daily life and to strengthen their positive mental health and wellbeing. The topics covered are: how the brain develops; how feelings, thoughts and behavior are linked together; common thinking styles; dysfunctional thinking; and coping strategies. Between the sessions the students had homework practicing some of the coping strategies. Strategies which are common in cognitive behavior therapy, such as filling out forms (ABC-model) and reflect on and dispute irrational thoughts.

MindPower is rooted historically and scientifically in the well-tested Coping With Depression Course (CWD) [22–19, 25]. However, MindPower differs from CWD in several ways. While, CWD courses usually are delivered to participants who are at least mildly depressed, or at risk of developing depression, MindPower is delivered universally to all students independently of their mental health challenges. While CWD courses are usually delivered in a health care setting, MindPower is delivered in a classroom setting during ordinary school hours. While CWD courses traditionally are delivered by psychologists, nurses or other health professionals, MindPower is delivered by trained high school teachers of varied professional background. Those who teach practical vocationally-oriented student programs, may be electricians, plumbers, engineers etc. Those who teach theoretically-oriented programs aimed at further academic studies, may be teachers in mathematics, biology, language, social science etc. Furthermore, MindPower is worded in a language reflecting “everyday challenges” (e.g. “discouraged”, “distressed”) rather than in “illness” or “disorder” terms (e.g. “depression”, “anxiety”). Common to most of the CWD-courses, including MindPower, is that they are organized in eight weekly sessions and two booster sessions. However, while in the CWD courses each session lasts for 2.5 h, each MindPower session is adapted to the school schedule and lasts for 1.5 h.

Before the start of the study, approximately 170 high school teachers attended a five-days, group based, intensive training course in how to teach MindPower. In addition to theoretical lectures, guidelines and instructions, the course included role playing and homework. The main modules consisted of learning about how the adolescent brain works and develops, and overall models in cognitive behavior theory. Training and certification were arranged by the organization ‘Fagakademiet’.

The coordinator of the study together with the designer of the MindPower version of the CWD course, Trygve Børve, met all the teachers. The teachers received information about the purpose of the study, study design and the coordinator’s phone number and e-mail address in case they had questions about the study. The teachers provided the information sheet and consent form to the students and posted the consents back to the coordinator along with students’ contact information in an encrypted file. There were a 100 percent coverage of consents. Only adolescents with valid consent from parents (15 years of age) were included in the study (78 percent). Participants who were 16 years of age responded on the consent in the online questionnaire (22 percent). In addition to participating in the training course, the teachers were encouraged to gather regularly in meetings to discuss experiences of teaching MindPower. Prior to each data collection, the teachers received a short list of key points to ease remembering of what to do. The list contained the date for the next data collection, and some questions they might ask themselves: “According to the study design, have I calculated time in class for responding on the questionnaire?”, “Did I remember to administer the questionnaire at the start of the session and not at the end of the class?”, “If there are any changes, have I e-mailed the coordinator?” and “Did I remember to give myself an applause for all the work I have done with assisting the students in responding to the online questionnaire?”.

### Statistical analyses

The data was analyzed with linear mixed models and paired samples t-tests, using R 4.0.2 [45] and SPSS 26 [46].

Linear mixed models are statistical models that contain both fixed and random effects and can handle non-balanced data with missing entries and repeated observations [47, 48].

In total, eight linear mixed models were fitted for each of the two outcome variables, depression and anxiety (SCL-8) and depression (RADS-2:SF). Model 1, serving as a baseline reference model, included only fixed and random intercepts. This allowed the dependent variable to vary across participants, but not across time. In subsequent models, the following components were added; a fixed effect of time (model 2), a random effect of time (model 3), a random effect of school (model 4), and an indicator variable for intervention group membership (model 5).

In the mixed models, the effect of the MindPower course is tested by the coefficients “Course”, and “Time*Course”, the interaction between time and the completion of MindPower. A change in symptom levels over the MindPower course, beyond what can be accounted for by the linear effect of time is in model 6 captured by the estimate of the regression coefficient for an indicator variable marking the completion of the course. The variable “course” is coded “0” until T2 and T4 for the IG1 and IG2 respectively, after which point it is coded “1”.

In model 7, a change in the fixed effect of time on depressive symptoms after the course is completed, is captured by the interaction between time and Course. A consequence of this parametrization is that once the interaction coefficient is included, “Time” quantifies the change “pre course completion”.

Finally, in model 8, a coefficient capturing the effect of male gender is added to the model y.

In all analysis, there are controlled for clustering within individuals and within schools.

## Results

Table 1 shows mean scores, n and standard deviations (SD) on SCL-8 and RADS-2:SF across the seven MindPower measurement points. The table also lists SCL-8 scores in the UngData reference sample; Students that participated in MindPower, and students that did not participate in MindPower.

The depression and anxiety mean scores (SCL-8) in Table 1 show that before the start of MindPower (T1), the SCL-mean scores in IG1 were very different from IG2, but both IG1 and IG2 were under the cut-off at 1.95 at T1, (1G1 = 1.76 and IG2 = 1.89), indicating no or minimal symptoms of anxiety and depression. In IG1 there was an increase from the beginning (1.76 at T1) to the end of the eighth week of the program (1.88 at T2), no change at the booster sessions (T3 and T4), a slight increase at T5, and almost no change at T6. The mean scores at T6 are still indicating low levels of anxiety and depression, and very similar to the mean score of UngData (1.99). In IG2 there was almost no change before the assessment, a slight increase from the beginning (1.90 at T3) to the end of the eighth week of the program (2.00 at T4), and a slight increase at the first booster session (T5). But at the second booster session (T6), the mean was below the initial mean at T1.

The depression mean scores (RADS-2:SF) in Table 1 also show, before the start of MindPower (T1), the total -scores in IG1 and IG2 were very different, but both were under the cut-off at 26 (IG1 = 18.83 and IG2 = 19.70), indicating normal range of symptom endorsement, and below clinical levels. The total scores ended higher in IG1 from T1 to T6 (T6 = 21.15) and slightly lower in IG2 from T1 to T6 (T6 = 19.92). The mean scores at T6 are still indicating low levels of anxiety and depression. This indicates that overall the MindPower-sample was within the normal range of symptom endorsement. Mixed models are a flexible approach that makes it possible to utility all the data. We found dropout to be associated with age and gender (the older male youths being overrepresented). But those who dropped out, did not differ from complete responders on their last measurement of either SCL-8 or RADS-2:SF (all *p* > 0.05), suggesting that there was no systematic dropout associated with the outcome variables of interest. Dropout was modest over the first three time points, but increased subsequently. At T7, only 81 responded to the final questionnaire. Only 11 out of 1673 students responded to the questionnaires seven times on SCL-8; 95 out of 1673 students responded six times. To RADS-2:SF, only 12 students responded seven times, and 100 students responded six times.

Scores on depression and anxiety (SCL-8, orange) and depression (RADS-2:SF, blue) across the seven measurement waves, standardized with respect to measures at baseline. Results from IG1 are given on the left, and IG2 on the right. The colored region indicates the eight weeks during which the group participated in the active part (eight weeks course) of the MindPower program./////

**Fig. 2:**
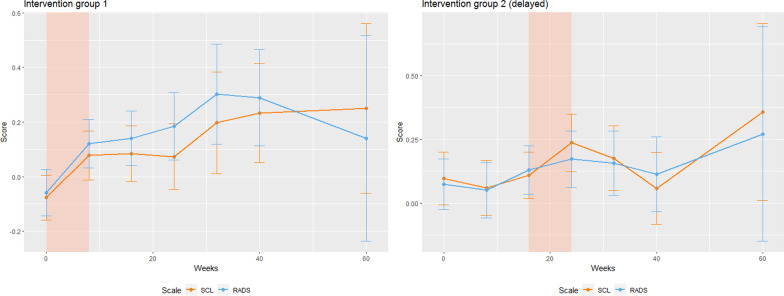
SCL-8 and RADS-2:SF across the seven measurement waves

The timeline in Fig. 2 shows that baseline starts at zero weeks (T1) and the data collection ends after 60 weeks (T7). Intervention group 1 is significantly lower than intervention group 2 at baseline on both SCL-8 and RADS-2:SF. There was a significant increase in SCL-8 and RADS-2:SF at the next time point (T2) in IG1. Whereas IG2 functioned as a control group and experiences a slight decrease. There was a non-significant increase in IG1 after the program. Furthermore, there was an increase from the second intervention group started the program, and a significant decrease in RADS-2:SF (SCL-8 was not significant) after the program. At the follow-up, 60 weeks after baseline, the n was low and not valid.

**Fig. 3:**
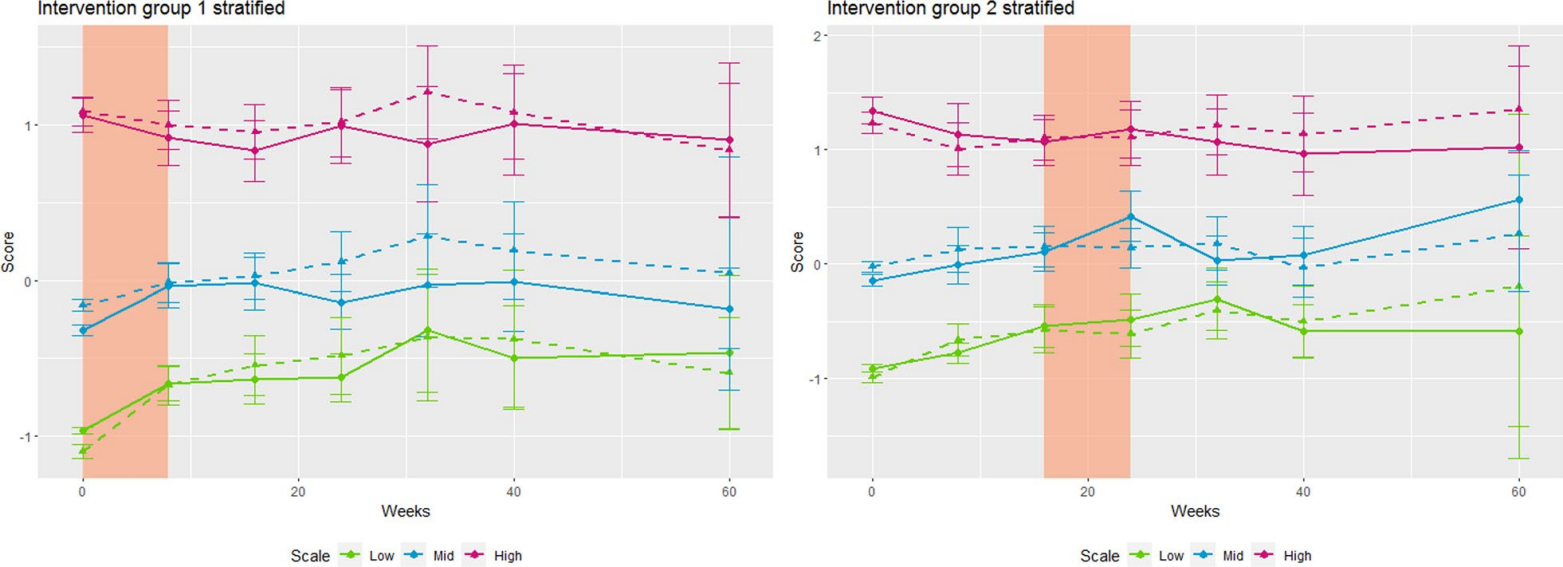
Scores on SCL-8 and RADS-2:SF across the seven measurement points

The highlighted time intervals in both Fig. 2 and 3 represent the period during which a given group underwent the MindPower course (week 0–8 in IG1, and 16–24 in IG2). Furthermore, the error bars represent 95% confidence intervals.

The scoring on the y-axis is the same in Fig. 2 and Fig. 3, and represents units standardized with respect to wave 1, i.e. $mean ({(SCL}_{i}-{mean}_{ wave 1})/{sd}_{wave 1})$. In Fig. 3 the sample is stratified into three groups representing 33% percentiles at wave 1. Results from IG1 are given on the left side, and IG2 on the right. The scores show the level of symptoms (high, medium, low). Scores on depression and anxiety (SCL-8, solid) and depression (RADS-2:SF, dashed) across the seven measurement points, stratified by and standardized with respect to scores at baseline (T1). The colored region indicates the eight weeks during which the group participated in the active part of the MindPower program.

Figure 3 illustrates the depression levels across the seven waves stratified by scores at T1. Here we can see that the increase in anxiety and depression levels across the study period is almost entirely attributable to increases in the low or middle group.

Table 2 shows the fixed coefficients from eight linear mixed models with SCL-8 as the dependent variable. Compared to the baseline model (model 1) which only includes a random intercept, adding a linear effect of time (model 2) was found to lead to a significant improvement in fit (-2∆LL = 24.428, p < 0.001). Subsequently adding random effects of time (model 3) and schools (model 4), also both resulted in a significant improvement (-2∆LL = 83.354, p < 0.001) and (-2∆LL = 5.872, p = 0.015), respectively. The estimate of Group differences in model 5, indicates that the differences between IG1 and IG2 observed at baseline are significant, despite our randomization (-2∆LL = 5.488, p = 0.019). Adding an effect representing change in SCL-8 score over the eight-week course period (model 6), improves fit beyond what can be accounted for by the overall linear trend (-2∆LL = 5.03, p = 0.025).

**Table 2:** Estimates from linear mixed models of SCL-8 scores across the seven measurement points

	Dependent variable: SCL-8							
	[1]	[2]	[3]	[4]	[5]	[6]	[7]	[8]
Time		0.003^***^	0.003^***^	0.003^***^	0.003^***^	0.002^*^	0.003	0.002^*^
		(0.001)	(0.001)	(0.001)	(0.001)	(0.001)	(0.002)	(0.001)
Group					0.087^**^	0.090^**^	0.096^**^	0.097^***^
					(0.037)	(0.037)	(0.038)	(0.035)
Course						0.055^**^	0.041	0.053^**^
						(0.024)	(0.030)	(0.024)
Time*Course							− 0.002	
							(0.002)	
Male								− 0.433^***^
								(0.035)
Constant	1.887^***^	1.888^***^	1.888^***^	1.916^***^	1.873^***^	1.839^***^	1.854^***^	2.029^***^
	(0.018)	(0.018)	(0.018)	(0.041)	(0.044)	(0.047)	(0.050)	(0.039)
Observations	4577	4577	4577	4577	4577	4577	4577	4577
Log likelihood	− 4525.296	− 4513.082	− 4471.405	− 4468.469	− 4465.725	− 4463.210	− 4462.880	− 4389.644
Akaike Inf. Crit	9056.592	9034.164	8954.809	8950.939	8947.450	8944.419	8945.761	8799.289
						*p < *0.05*	**p < *0.01*	***p < *0.001*

However, the interaction between time centered at the last day of the course and course completion was not significant (model 7), and adding the interaction also resulted in an increase in AIC, compared to model 6. This suggests that there was no significant change in the trajectory of SCL-8 following completing the MindPower course. This interaction was subsequently discarded from the model. Lastly, we observed a clearly significant sex-difference in SCL-8 scores, with boys scoring 0.433 units lower. In addition to having a significantly better fit than model 6, under which it is nested, model 8 also had the overall lowest AIC score.

Estimates from the same models for the RADS-2:SF outcome variable are given in Table 3. The results are very similar to the SCL-8 findings. Also for the RADS-2:SF variable, model 8 was found to have the best fit. Also for RADS-2:SF we observed a small but significant increase throughout the study period, a significant baseline difference between IG1 and IG2, an increase in scores over the course period, and a markedly lower RADS-2:SF score among boys.

**Table 3:** Estimates from linear mixed models of RADS scores across the seven measurement points

	Dependent variable: RADS:SF-2							
	[1]	[2]	[3]	[4]	[5]	[6]	[7]	[8]
Time		0.003^***^	0.004^***^	0.004^***^	0.004^***^	0.003^***^	0.003	0.003^***^
		(0.0005)	(0.001)	(0.001)	(0.001)	(0.001)	(0.002)	(0.001)
Group					0.049	0.051	0.051	0.055^*^
					(0.031)	(0.031)	(0.031)	(0.030)
Course						0.037^*^	0.038	0.036^*^
						(0.021)	(0.025)	(0.021)
Time*Course							0.0002	
							(0.002)	
Male								− 0.250^***^
								(0.030)
Constant	1.999^***^	2.000^***^	1.999^***^	2.022^***^	1.997^***^	1.974^***^	1.951^***^	2.087^***^
	(0.015)	(0.015)	(0.015)	(0.034)	(0.036)	(0.038)	(0.030)	(0.037)
Observations	4647	4647	4647	4647	4647	4647	4647	4647
Log Likelihood	− 3844.872	− 3819.545	− 3787.441	− 3783.601	− 3782.369	− 3780.746	− 3784.567	− 3746.798
Akaike Inf. Crit	7695.744	7647.090	7586.882	7581.201	7580.738	7579.492	7589.133	7513.596
						*p < *0.05*	**p < *0.01*	***p < *0.001*

## Discussion

We tested an upscaled version of the Coping With Depression/Coping With Strain Course (CWD/CWS), called MindPower.. The traditional CWD/CWS courses have mainly targeted high risk groups for depression and been delivered within a health service context. The MindPower version differs from previous versions by being upscaled to a universal program and being delivered class wise to high school students during ordinary school hours, independently of their mental health status. In spite of classical recommendations to go for universal preventive interventions when a health problem is widespread [30], to our knowledge, nobody has tried to test the delivery of a course in the CWD/CWS-family universally since Clarke and coworkers in 1993 [22].

To test the efficacy of MindPower, we used a two-groups delayed intervention design combined with linear mixed models statistical analyses. To indicate whether the absolute symptom levels in the two intervention groups before, during and after the intervention differed significantly from the relevant general population, we compared the results with results from a large-scale population survey covering all students in the same age group and catchment area (UngData) [41].

We measured symptom levels of anxiety and depression with SCL-8 in order to be able to compare our results with the relevant general population, and with RADS-2:SF, since this instrument is well tested and shown to have good psychometric properties, is adapted particularly to this population, and is assumed to be sensitive to change [42].

The monitoring test showed that only half of the students in both intervention groups reported to have completed the tenth and last MindPower session. This deviates from the instructions in the study design and the course leader manual. All the ten sessions are regarded as necessary, because it takes time to learn all the content in MindPower. Less than complete implementation may attenuate effects of the intervention seriously.

The findings indicate no reduction or preventive effect on the anxiety and depression levels of the students. The main findings show, however not conclusively because of the limitations of this study, a small but significant symptom increase during the eight weeks course period, and throughout the study period. This slight increase in self-reported symptom levels of anxiety and depression in the MindPower sample was almost entirely attributable to those who at start of the study had the lowest symptom scores, which again were markedly lower than the scores of their peers in the comparable UngData population sample. Boys had significantly lower levels of depression and anxiety than girls throughout the MindPower study period. This is consistent with repeated reports that girls tend to have an elevated level of common mental distress in the general population as compared to boys [41].

### Interpretation

Why did we not find positive effects of MindPower on symptoms levels of anxiety and depression?

The most obvious explanation is that a reduction of symptom levels among these participants would be hard to achieve because most of the students before the start of the study had only moderate or low levels of symptoms and even lower than a comparable group from the general population of peers (UngData).

Another possible explanation is selective dropout. The high dropout across time in this study leads to loss of statistical power and possibly also patterns of selection which again hampered our conclusions. The dropout was associated with age and gender; the older male students being overrepresented.

However, those who dropped out, did not differ significantly from complete responders on their last measurement of either SCL-8 or RADS-2:SF (all *p* > 0.05). Drop out was not systematically associated with the outcome variables of interest. If the intervention had had a significantly symptom effect in the two groups, such an effect should have been revealed in spite of the high level of dropout.

In fact, although modest, we saw a slight increase of symptom levels across time. Since the students seem to be a selected group with lower than peer population levels of symptoms, this may be due to statistical regression. In that case, the increased symptom levels were not due to MindPower, but to statistical effects.

However, it is not unusual that when measures of levels of anxiety and depression are repeated in non-clinical populations, the level of self-reported symptoms increases. This has been attributed to destigmatizing and to increased awareness.

This study included seven measurement points, all with the same symptom measures, across more than one year in a group of participants with little previous experience in reporting on their mental health. This may have facilitated a small awareness effect. The fact that the students during the project period were trained in how to deal with issues of life, may also have facilitated such an effect [49]. From a mental health literacy point of view, increased awareness of one’s state of mental health may be regarded as a positive result.

Although several of these explanations may have been operating, we cannot exclude the possibility that the results turned out as they did because MindPower, as it was implemented, did not meet the students’ expectations or for other reasons was ineffective. It might be that the program is not effective in reducing depressive symptoms in adolescents in a high school setting.

In that case, however, at the end of the project, the levels of anxiety and depression were still low and not higher than the level found in the comparable population study among students who had not received any intervention. Consequently, although a slight symptom increase was observed, it can hardly be argued that the intervention was harmful.

### What can we learn from this study?

This study was launched and conducted in a very positive contextual atmosphere. The Norwegian government had pledged that all schools must provide life-skills education. A new national curriculum plan on how to include public health and life skills in the schools was in the process of being launched. Researchers, students, teachers and politicians had for a long time argued for equalization of mental and physical health in the school. Openness about mental health issues had increased significantly and continuously since The Ten Years National Task Force On Mental Health (1999–2008).

In the case of this study specifically, the top administrator of education in the county welcomed the study and was hands on, enthusiastic, and effective in supporting communication and collaboration with the school administration and the schools. The Deputy Minister of Health of Norway visited the project, which was also mentioned in the text of the National Budget (2020). Several motivation meetings with the teachers before and during the project period indicated high motivation among school leaders, teachers, and students to participate.

Comprehensive preparations were laid out before implementation. All the involved teachers received intensive training, arranged by the organization ‘Fagakademiet’. This included theoretical lectures, study design, practical guidelines and instructions, role-playing, and homework, ending in certification to teach MindPower. In an internal evaluation from Fagakademiet, the teachers reported very high satisfaction regarding the training. Prior to each data collection, the teachers received a short list of key points to ease remembering of what to do. In addition, all 170 teachers received the principal researcher’s phone number and e-mail address in case they had questions about the study.

With such a positive context and such a thorough implementation and enthusiastic follow up, what could have been done to improve intervention implementation and data collections? A qualitative study has addressed this question [43]. In addition, we have collected information through a number of informal sources. This has provided us with several learning points for others who intend to launch large-scaled universal mental health initiatives among high school students.

The first problem identified was that, at some schools, the teachers did not have sufficient support from their school administration. In particular, some lacked assistance from their co-teachers to adapt the MindPower program into the curriculum.

A solution was, as some schools did, to establish a forum at the school that could include mental health personnel. Their intention was to share positive and negative experiences and to seek advice and support.

Another problem is lack of clarity with regards to teachers’ job descriptions. Teachers questioned whether they alone should have the responsibility of reducing symptoms of anxiety and depression among adolescents. Furthermore, they questioned whether school teachers have the mandate, the skills and the resources needed in order to effectively promote students’ mental health. Other teachers maintained that teaching mental health is not a school-teacher’s job. Teaching and promoting students’ mental health is rather the job of the health services.

Solutions to this kind of challenges could be that the program leadership and principals together give clear directions on these concrete issues. This must be sorted out well before the teachers go through comprehensive training to become competent life-skill teachers. However, relevant authorities had not provided clear mandates and descriptions with regards to how life-skills should be implemented in schools, and what to expect from the teachers.

Based on experiences from the present study, we have come to the conclusion that teachers have not been sufficiently well mandated and resourced for the task of taking the lead in implementing and conducting school-based life skills training programs. By teaching life-skills, the teachers alone cannot be expected to reduce anxiety and depression levels among adolescents. Instead, life skills can be taught in terms of “how life is”, implying that challenges in life are normal and that no one is alone in experiencing such feelings. In this way one might be better off in approaching such challenges in life.

A third problem is that teachers were instructed to participate in the preparatory course, whether they were motivated or not. Some teachers were not comfortable handling issues related to emotions. In some schools, the school administrations did not allow teachers to choose not to teach MindPower.

To address this, a solution is to only admit motivated teachers to teach life-skills programs. Unmotivated teachers are seldom good teachers. Consequently, as far as possible, teachers who see this as a natural part of their day-to-day practice as a teacher should perform life-skills education. It is necessary to respect that it cannot be mandatory to teach life-skills. On the other hand, some teachers maintained that all teachers are practicing life-skills education with their students anyhow when they are communicating with students. An example is mobilizing self-efficacy by teaching the students how to overcome their fear of making mistakes in math.

A fourth problem may be that the MindPower program has not been sufficiently well designed and tailored for classroom-based educational purposes. Teachers experienced considerable difficulties running the MindPower program in the classroom.

A solution might be to involve teachers and students in a process of program revision. Ideas and opinions of teachers and students are crucial in order to succeed. However, in the present project, students and teachers were asked to give feedback on the course book before the implementation of the program. Furthermore, MindPower lasts for 90 min rather than two and a half hours as in traditional Coping With Depression/Coping with Strain-courses [23, 24]. This change was made by the designer of MindPower to ease the implementation of MindPower in schools.

The fifth problem is general implementation issues. Adequate implementation requires strengthening the program on several dimensions. During the program implementation in schools, it turned out that the teachers had deviated from the implementation plan on several points, for instance by not completing all the ten MindPower sessions, and by shortening the 90 min sessions. It turned out to be too time consuming and for practical reasons not easy to fit into the curriculum. The implementation varied considerably across schools. The ten schools had different needs and different curricula, and therefore had to develop their own local implementation plans to make the implementation possible. A demotivating factor for the students was when their self-selected favorite course was replaced with MindPower.

Solutions for this issue, is to test for fidelity. The first author of this study did not receive the necessary approval from the first author’s research institute to administer a questionnaire on monitoring fidelity.

There are at least four major problems that should have been addressed in a fidelity test, as described above, and confounding variables affect the findings of the study. To which extend the course book and the course leader manual have been used according to intent, affect the results. It is essential to test whether the 170 course leaders followed the course leader manual, the course book and the study design. Otherwise we cannot know whether we measure the depression and anxiety effects of the MindPower course, or something else.

It takes time for the students to learn and do the practical assignments in life-skills training, and it is essential to make the time and effort to learn life-skills [49].

Another problem was difficulties with data collections. The students had difficulties in retrieving the questionnaire due to technical issues. There were difficulties in administering the questionnaires with the correct e-mail addresses, and the participants had difficulties in both retrieving and opening the questionnaires at the seven time points. An additional problem in this study was that after we had received the student e-mail addresses directly from the teachers, during the preparations for the data collections, these e-mail addresses were replaced with less updated addresses retrieved from The Common Contact Register of Norway. Consequently, a significant portion of the eligible students did not receive the initial invitation to participate.

This issue has several solutions. It is necessary that the technical partner, which is administering the data collections, accepts the plan for what to do if there are technical failures. Such a plan includes making additional links with the questionnaires, which can be sent to the participants and teachers if technical problems occur. Pretesting data collection procedures is important, including making sure you have the best possible records of e-mail addresses and telephone numbers of study participants.

### Strengths and limitations

This study, like all studies has its strengths and weaknesses. Strengths include the adapted randomized control trial design (RCT), with inclusion of a delayed intervention group, which functioned as a control group until the intervention was implemented. For ethical reasons, it is good to let all study participants benefit from the intervention, only with a period of delay of four months before the intervention is implemented in the second group. Compared with a pure RCT design, the two-group design with a delayed intervention in one of the groups may be less strong. Still the latter design has several strengths. If a pattern of stability before the intervention in the second group is observed, followed by a decline in depression and anxiety during and possible after the interventions in the second group and a similar pattern during and after the intervention is observed in the first group, we have strong indications of program effects.

In the context of the present study, also, data from the UngData study made it possible to compare outcome variables with corresponding prevalence and means estimates of the general population. Further, utilization of mixed models statistical analysis, with its handling of missing with maximum likelihood estimation contributes to making results less biased. The present study is based on relatively large numbers of participants, over 1600 in MindPower and over 18000 in UngData. The outcome measures used in our study are high quality instruments commonly utilized in studies on adolescents. Furthermore, when two such measures are administered repeatedly, changes over time can be examined.

Results presented above show that mean scores on outcome measures show the same patterns of change in both groups. This strengthens our trust in the findings in this study.

Limitations include insufficient fidelity assessment. Beyond systematic reports from students, there are only verbal reports and sporadic emails from teachers revealing problems of implementation and data collections that occurred during the study. Also, during the electronic data collection serious problems were encountered. Due to problems with the list of e-mail addresses, questionnaires often did not reach students. This may have contributed to attrition and selection bias, even at the first measurement occasion. This might explain why we observed significant differences between the two intervention groups already at baseline. Teachers as well as students reported problems with retrieving the questionnaire as stressful and time consuming, which caused a bad teaching situation during implementation of the MindPower program [50]. Furthermore, even though analyses indicate that drop-out did not produce the observed increases in anxiety and depression levels, a limited number of participants filled in and submitted the questionnaire on all 7 data collection occasions. In this study, the mean score from the follow-up (T7) is not sufficient, because of the low participation at this last data collection. Furthermore, the lack of further data collections (after M7) makes it impossible to examine more long-term preventive effects of MindPower on depression and anxiety. The findings might not have generalizability if the school-samples e.g. background variables, teacher training, organization and school structures, differs in great extend from the current samples.

Although several studies have found positive effects on youth mental health after exposure to life-skills programs, reviews have concluded that there are serious risks of biases in a number of previous studies. This cast doubt on conclusions from these studies [51–53]. Therefore, there is limited evidence that educational setting-based interventions focused solely on the prevention of depression or anxiety are effective [47]. However, in this paper we have provided a number of advises for future research on universal mental health promotion and prevention projects in order to improve implementation as well as data collection quality.

## Conclusion

In the present study, no intervention effects of the MindPower program implemented in schools in one Norwegian county were found. The level of symptoms in MindPower were at the same level as in the relevant comparison population (UngData) after the trial. Also at the end of the study, the mean symptoms were low. There were only small changes over the timeline from start to end of the study.

The study, however, suffers from problems. Implementation fidelity was not allowed to be examined. Less systematic evidence from oral and e-mail communication with teachers and students indicate that the intervention was not implemented as planned. There were also problems with data collections and a high level of drop-out of participants.

Beyond problems with the intervention, problems with the monitoring of fidelity of the intervention and problems with data collections, there are additional reasons for lack of intervention effects. A reduction in anxiety and depression levels may have been difficult to obtain because of the moderate to low levels of symptoms at baseline.

There was a slight significant increase in the levels of anxiety and depression throughout the study period, however, the increase is entirely attributable the students with the lowest symptom scores at baseline. This is interpreted as a regression effect, and the increase is not caused by the MindPower intervention. Advices for future studies based on experiences from the present study may be valuable for improving the quality of future studies.

## Data Availability

The datasets analyzed during this study is not publicly available because of ethical restrictions. Contact the corresponding author.

## Abbreviations

CWS: Coping with strain course
CWD: Coping with depression course
IG1: Intervention group 1
IG2: Intervention group 2
NIPH: Norwegian institute of public health
NOVA: Norwegian social research
